# Upregulated GBP2 exacerbates Parkinson's disease pathogenesis by impairing NIX-dependent mitophagy

**DOI:** 10.1016/j.redox.2026.104029

**Published:** 2026-01-17

**Authors:** Wenqi Cui, Tianlu Wang, Juan Feng

**Affiliations:** a Department of Neurology, Shengjing Hospital of China Medical University, Shenyang, China; b Department of Neurology, The First Affiliated Hospital of Jinzhou Medical University, Jinzhou, China

**Keywords:** Parkinson's disease, Mitochondrial dysfunction, GBP2, NIX, Mitophagy, Geranylgeranylation

## Abstract

Parkinson's disease (PD), characterized by dopaminergic neuron loss, still lacks disease-modifying therapies due to incompletely understood mechanisms. Guanylate-binding proteins (GBPs) are well-known immune regulators, but their roles in PD are largely unknown. In this study, we identify GBP2 as a critical driver of PD pathogenesis by impairing mitophagy. We found that GBP2 was significantly upregulated in the substantia nigra of PD patients, and in both MPTP-induced and A53T transgenic mouse models, as well as in MPP^+^-treated or A53T α-synuclein-overexpressing SH-SY5Y cells. Both in vivo and in vitro, genetic knockdown of GBP2 robustly alleviated the MPTP/MPP^+^-induced motor deficits, dopaminergic neuron loss, and apoptosis. Mechanistically, PD-related stress promotes GBP2 geranylgeranylation, driving its mitochondrial accumulation. At mitochondria, GBP2 directly binds the mitophagy receptor NIX via its large GTPase domain and targets it for ubiquitin-proteasomal degradation, thereby suppressing NIX-mediated mitophagy. Accordingly, GBP2 knockdown enhanced mitophagy, improved mitochondrial homeostasis, and protected against neuronal apoptosis. The neuroprotective effects of GBP2 knockdown were abolished by either pharmacological inhibition of mitophagy or genetic knockdown of NIX, indicating a linear pathway. Importantly, therapeutically targeting geranylgeranylation with GGTI298 significantly attenuated MPTP-induced neurotoxicity. Our study unveils a novel, druggable axis in PD pathogenesis where GBP2 disrupts mitochondrial quality control. Targeting GBP2 geranylgeranylation with GGTI298 presents a promising therapeutic strategy.

## Highlights

• GBP2 upregulation in PD models exacerbates neurotoxicity and motor deficits.
• GBP2 accumulates in mitochondria, where it exacerbates mitochondrial dysfunction upon MPP^+^ stress.
• GBP2 binds to the mitophagy receptor NIX via its GTPase domain and promotes its ubiquitination, thereby facilitating NIX degradation and ultimately suppressing mitophagy.
• Geranylgeranylation is essential for targeting GBP2 to mitochondria, and its pharmacological inhibition by GGTI298 alleviates PD pathology.

## Graphical abstract

**Figure:**
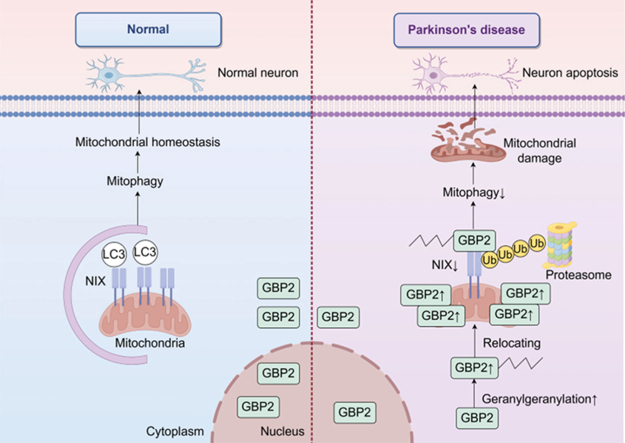

## 1 Introduction

Parkinson's disease (PD) is the most rapidly progressive neurodegenerative disorder worldwide, characterized primarily by the progressive loss of dopaminergic neurons in the substantia nigra [1]. Clinically, PD is characterized by a classic motor triad (bradykinesia, rigidity, and resting tremor) as well as a range of non-motor symptoms [2]. However, current PD treatments, including levodopa, provide only symptomatic relief and cannot halt or slow the underlying neurodegeneration. Thus, elucidating the molecular mechanisms driving PD pathogenesis is essential for developing disease-modifying therapies [3]. Mitochondria play a central role in PD neurodegeneration by regulating cellular energy production and signaling pathways that determine cell survival or degeneration based on bioenergetic status [4]. A key mechanism in mitochondrial quality control is mitophagy, a selective autophagic process that removes damaged mitochondria, serving as a critical defense for maintaining mitochondrial homeostasis and preventing neuronal loss [5]. Among key mitophagy regulators, Nip3-like protein X (NIX/BNIP3L), an outer mitochondrial membrane protein belonging to the BH3-only subgroup of the BCL2 family, represents an important alternative mitophagy pathway in PD [6]. It promotes mitophagy by facilitating autophagosome formation and recruiting them to damaged mitochondria [7]. NIX activity is tightly regulated by post-translational modifications, particularly phosphorylation and ubiquitination [8]. Although the PINK1/Parkin pathway is well-studied, it is frequently impaired by mutation in PD, underscoring the importance of alternative pathways like NIX-mediated mitophagy. However, regulation of NIX remains unclear, as its dysfunction typically stems from non-genetic causes. Thus, identifying upstream regulators controlling these pathways is crucial [9].

Guanylate-binding proteins (GBPs) belong to the dynamin superfamily of GTPases and are known to mediate diverse functions including immune regulation and host defense [10]. In mice, the 11 GBP genes (GBP1–11) are distributed across two chromosomal clusters, whereas humans possess seven GBP genes (GBP1–7) located within a single cluster on chromosome 1 [11]. Owing to its important roles in inflammation, antimicrobial immunity, and cancer, GBP2 has become one of the most widely studied members of the GBP family, as we previously reviewed [12]. GBP2 is composed of three principal structural domains (Table S1) [13,14]. Notably, the C-terminus of GBP2 contains a CAAX motif that undergoes geranylgeranylation, which is a key post-translational modification that enhances the protein's ability to associate with endomembrane organelles [15,16]. Preliminary evidence suggests that GBP2 is expressed in the nervous system, yet its functional role in neurological disorders remains largely correlative and derived from omics studies [[17], [18], [19]]. Whether GBP2 plays a direct causal role in PD pathogenesis, and through what mechanism, remains an open question. In support of this possibility, our bioinformatic analysis of transcriptomic data from the GEO database revealed marked upregulation of GBP2 and GBP6 in the substantia nigra of PD patients. Moreover, although GBP2 has been shown to suppress breast cancer cell invasion by regulating Drp1-mediated mitochondrial fission, whether it influences mitochondrial function and mitophagy remains unexplored [20]. These findings led us to hypothesize that GBP2 may play a non-canonical role in PD pathogenesis. Moreover, our preliminary screening identified the mitophagy receptor NIX as a putative mediator.

Based on these observations, we hypothesize that GBP2 contributes to PD pathogenesis by disrupting NIX-mediated mitophagy, a process that is modulated by the geranylgeranylation of GBP2 itself. To test this hypothesis, we aimed to: (1) definitively establish the expression of GBP2 in cellular and animal models of PD; (2) investigate the functional consequences of GBP2 knockdown on neuronal survival and mitochondrial integrity; (3) elucidate the underlying mechanism, focusing on its interaction with the mitophagy receptor NIX; and (4) explore the therapeutic potential of targeting GBP2 geranylgeranylation. Together, our results identify and validate a novel GBP2–NIX axis that critically regulates mitochondrial homeostasis in PD, nominating this pathway as a promising therapeutic target.

## 2 Materials and methods

### 2.1 Animal models

Male C57BL/6J mice (7–8 weeks old, weighing 20–25 g) were obtained from Beijing HFK Bioscience Co., Ltd. The animals were housed individually in plastic cages under a 12-h light/dark cycle with ad libitum access to food and water. Room temperature was maintained at 20–26 °C and relative humidity at 40–70 %. All mice were acclimated to the housing environment for at least one week prior to experiments. For GBP2 knockdown, an adeno-associated virus serotype 9 carrying GBP2 shRNA (AAV9-GBP2-shRNA; 1.48 × 10^13^ vg/mL) was purchased from Sangon Biotech. An AAV9 carrying a negative control shRNA (AAV9-shNC; 1.92 × 10^13^ vg/mL) was used as control. Mice received bilateral microinjections into the substantia nigra with 1 μL of either AAV9-GBP2-shRNA or AAV9-shNC. Stereotaxic coordinates relative to bregma were as follows: anteroposterior (AP), −3.2 mm; mediolateral (ML), ±1.2 mm; dorsoventral (DV), −4.3 mm from the dura. Injections were performed at a rate of 0.1 μL per minute, using coordinates derived from the mouse brain atlas [21]. After a 4-week recovery period to allow for viral expression and GBP2 knockdown, a PD model was induced by intraperitoneal injection of MPTP (30 mg/kg daily for five consecutive days; Sigma-Aldrich, Cat#M0896) [22]. Control mice received an equal volume of normal saline. To further examine the role of protein geranylgeranylation in this process, a subset of mice was treated with the geranylgeranylation inhibitor GGTI298 via intraperitoneal injection (12 mg/kg daily for fourteen consecutive days; MedChemExpress, Cat#HY-15871) [23].

The A53T transgenic mouse samples were obtained from another project within our research group. Purchased male B6; C3-Tg (Prnp-SNCA∗A53T) 83Vle/J mice were used as the F0 paternal generation and C57BL/6J mice as the maternal generation (Shanghai Model Organisms Center). Males and females were housed together in a 1:2 ratio for breeding. Neonatal mice were ear-tagged and tail-clipped within 7 days after birth for genotyping. All mice used in this study were male Tg mice from the same batch of the F3 generation and were sampled at 13 months of age.

All animal experiments were approved by the Ethics Committee of Shengjing Hospital of China Medical University (Shenyang, China; Ethics No. 2023PS1295K and 2022PS453K).

### 2.2 Cell culture and transfection

The human neuroblastoma cell line SH-SY5Y and the human embryonic kidney (HEK) 293T cell line were obtained from Procell (Cat#CL-0208 and Cat#CL-0005, respectively). Both cell lines were authenticated by STR profiling and tested negative for mycoplasma contamination. SH-SY5Y cells were cultured in DMEM/F12 medium (Procell, Cat#PM150312) supplemented with 15 % fetal bovine serum (FBS; Procell, Cat#164210) and 1 % penicillin–streptomycin (Procell, Cat#PB180120). HEK293T cells were maintained in high-glucose DMEM (Procell, Cat#PM150210) containing 10 % FBS and 1 % penicillin–streptomycin. Both cell lines were incubated at 37 °C in a humidified atmosphere with 5 % CO_2_. To establish an in vitro PD model, SH-SY5Y cells were treated with 1.5 mM MPP^+^ (Sigma-Aldrich, Cat#D048) for 24 h (a classical model for PD research), using a concentration optimized by CCK-8 assay [24,25]. To establish an in vitro A53T α-synuclein-overexpressing model, SH-SY5Y cells were transfected with a lentiviral plasmid encoding the human A53T mutant SNCA gene (Beijing Syngenbio Co). For knockdown of GBP2 and NIX expression, SH-SY5Y cells were transfected with specific small interfering RNAs (siRNAs) targeting GBP2 (si-GBP2) or NIX (si-NIX), using a non-targeting siRNA (si-NC) as a negative control. All siRNAs were supplied by HANBIO. Transfection was carried out using Lipofectamine 3000 reagent (Thermo Fisher Scientific, Cat#L3000015) following the manufacturer's instructions. The sequences of all siRNA oligonucleotides used are listed in Table S2.

### 2.3 CCK-8 assay

Cell viability was assessed using the Cell Counting Kit-8 (CCK-8; Apexbio, Cat#K1018) according to the manufacturer's instructions. Briefly, SH-SY5Y cells were seeded into 96-well plates at a density of 1 × 10^4^ cells per well and allowed to adhere for 24 h. The cells were then treated with various concentrations (0.5, 1, 1.5, 2, and 3 mM) of MPP^+^ for 24 h. After treatment, 10 μL of CCK-8 reagent was added to each well, and the plates were incubated at 37 °C for 4 h. Absorbance was measured at 450 nm using a microplate reader. Cell viability was expressed as a percentage relative to the control group.

### 2.4 RT-qPCR

Total RNA was extracted from cells and tissues using TRIzol reagent (Vazyme, Cat#R401). Complementary DNA (cDNA) was synthesized from 1 μg of total RNA with HiScript III RT SuperMix for qPCR (Vazyme, Cat#R323). Quantitative PCR was carried out on an ABI 7500 Real-Time PCR System (Thermo Fisher Scientific) using ChamQ Universal SYBR qPCR Master Mix (Vazyme, Cat#Q711). Each reaction was set up in a total volume of 20 μL, containing 10 μL of 2 × Master Mix, specific primers, and cDNA template. The thermal cycling protocol consisted of an initial denaturation at 95 °C for 30 s, followed by 40 cycles of 95 °C for 10 s and 60 °C for 30 s. The relative expression of target genes was calculated by the 2^(–ΔΔCt) method and normalized to GAPDH expression. All primer sequences used are provided in Table S3.

### 2.5 Western blot analysis

After washing with cold phosphate-buffered saline (PBS), cells were lysed with RIPA lysis buffer (Epizyme, Cat#PC101) containing 1 % protease inhibitor cocktail (Epizyme, Cat#GRF101) and 1 % phosphatase inhibitor cocktail (Epizyme, Cat#GRF102). Protein concentrations were determined using a BCA protein assay kit (Epizyme, Cat#ZJ102). Equal amounts of protein were separated by SDS-PAGE and transferred to PVDF membranes. The membranes were blocked with 5 % skim milk in TBST for 2 h at room temperature to minimize nonspecific binding. Subsequently, the membranes were incubated with specific primary antibodies diluted in Universal Antibody Diluent (Epizyme, Cat#PS119) at 4 °C overnight. After three 10-min washes with TBST, the membranes were incubated with horseradish peroxidase (HRP)-conjugated secondary antibodies (Table S4) diluted in TBST for 1 h at room temperature. Following another round of TBST washes, protein bands were visualized using a chemiluminescence detection system. The antibodies used are listed in Table S4.

### 2.6 Co-immunoprecipitation (Co-IP)

Co-IP assays were conducted to examine protein–protein interactions. In brief, cells were collected 48 h after transfection with the indicated plasmids and lysed in IP lysis buffer (Beyotime, Cat#P0013) containing protease inhibitors on ice for 30 min. After centrifugation at 14,000×*g* for 10 min at 4 °C, the protein concentration of the supernatant was measured by BCA assay. For each immunoprecipitation, 500 μg of total protein was incubated with the appropriate primary antibody or corresponding control IgG with gentle rotation overnight at 4 °C. Then, 40 μL of pre-washed Protein A/G Agarose beads (Beyotime, Cat#P2055) were added to the mixture and incubated for another 3 h. The beads were collected and washed three times with cold lysis buffer. Bound proteins were eluted by boiling in 2 × SDS loading buffer and subsequently detected by Western blotting using the indicated antibodies.

### 2.7 Ubiquitination analysis

To examine whether GBP2 regulates the ubiquitination of endogenous NIX, HEK293T cells were co-transfected with a plasmid encoding HA-ubiquitin (HA-Ub) along with either a Flag-GBP2 expression plasmid or an empty pcDNA3.1 vector (as control), using Lipofectamine 3000 (Thermo Fisher Scientific, Cat#L3000015). Forty-eight hours after transfection, cells were treated with 10 μM MG132 for 6 h to stabilize ubiquitinated species. Cells were then lysed and the lysates subjected to Co-IP using an antibody against endogenous NIX. Ubiquitination levels of endogenous NIX were assessed by immunoblotting the immunoprecipitates with an anti-HA antibody.

### 2.8 Immunofluorescence staining

Cells grown on glass coverslips were fixed with 4 % paraformaldehyde for 15 min at room temperature. After washing with PBS, cells were permeabilized with 0.1 % Triton X-100 (Solarbio, Cat#T8200) in PBS for 10 min, followed by blocking with 5 % bovine serum albumin (BSA; Solarbio, Cat#SW3015) for 1 h at room temperature. Cells were then incubated with appropriate primary antibodies diluted in 5 % BSA overnight at 4 °C in a humidified chamber. After three PBS washes, cells were incubated with fluorescent-labeled secondary antibodies diluted in 5 % BSA for 1 h at room temperature in the dark. Nuclei were counterstained with DAPI (Solarbio, Cat#C0065), and coverslips were mounted using anti-fade mounting medium. Fluorescence images were captured using either a widefield fluorescence microscope or a confocal laser-scanning microscope, as indicated in the figure legends. Specific microscope models and objective magnifications used are provided in the corresponding figures and legends. All image processing and analysis, including channel merging and intensity adjustments, were performed using ImageJ software (National Institutes of Health, USA).

### 2.9 Behavioral testing

Behavioral tests were performed 7 days after the last MPTP injection. In the open field test, mice were acclimated to the testing room for 30 min before being individually placed in the center of a square arena (40 cm × 40 cm). Their locomotor activity was recorded for 10 min using an automated video tracking system (EthoVision XT17, Noldus). The total distance traveled during the entire session was automatically calculated by the software for each animal.

For the rotarod test, mice were pretrained on the rotarod apparatus for five consecutive days before formal testing. During the test, each mouse was placed on a rotating rod that accelerated from 4 to 40 rpm over a 5-min period. The latency to fall off the rod was recorded. The final score for each mouse was calculated as the mean latency across three independent trials, with 10-min rest intervals between trials.

In the pole test, a wooden pole (50 cm high, 1 cm in diameter) wrapped with gauze to prevent slipping was placed vertically in the home cage, with a wooden ball (2.5 cm in diameter) attached to the top. Mice were acclimated to the setup for three days prior to testing. During the test, each mouse was placed head-up at the top of the pole, and the time taken to turn completely downward and descend to the base was recorded as the descent time. Each mouse performed three trials with 10-min inter-trial intervals, and the average time was used for analysis.

Blinding was maintained throughout data acquisition and analysis. The experimenter performing behavioral tests knew only each animal's randomized ID number. A second blinded researcher analyzed all data using these anonymized IDs. The group allocation key was securely held and was not revealed until after all analyses were complete.

### 2.10 Immunohistochemistry (IHC)

IHC was performed on paraffin-embedded mouse brain sections using an UltraSensitive SP IHC Kit (MXB Biotechnologies, Cat#KIT-9701) according to the manufacturer's instructions. Briefly, sections were deparaffinized in xylene and rehydrated through a graded ethanol series. Antigen retrieval was conducted by heating the sections in sodium citrate buffer. Endogenous peroxidase activity was blocked by incubation with 3 % hydrogen peroxide for 15 min. After washing with PBS, the sections were blocked with the kit-provided blocking serum for 30 min at room temperature. The sections were then incubated overnight at 4 °C with the following primary antibody: rabbit anti-TH (1:500, Abcam, Cat#ab137869). After washing, the sections were incubated with the kit-provided biotinylated secondary antibody for 30 min, followed by streptavidin–peroxidase complex. Immunoreactivity was visualized using 3,3′-diaminobenzidine (DAB) as the chromogen. The DAB reaction was stopped by rinsing the slides in distilled water. Finally, sections were counterstained with hematoxylin, dehydrated, cleared in xylene, and mounted with a synthetic mounting medium. Stained sections were examined under a light microscope, and representative images were captured.

### 2.11 Detection of apoptosis by flow cytometry and TUNEL staining

Cell apoptosis was quantified by flow cytometry using an Annexin V-FITC/PI Apoptosis Detection Kit (Seven, Cat#SC123). Briefly, SH-SY5Y cells were seeded in 6-well plates. After 24 h, cells were transfected with the indicated siRNAs. Following another 24-h incubation, cells were treated with MPP^+^ or PBS (control) for 24 h. Cells were then harvested by trypsinization without EDTA, washed twice with cold PBS, and resuspended in 1 × Binding Buffer. According to the manufacturer's protocol, the cell suspension was incubated with Annexin V-FITC and propidium iodide (PI) for 15 min at room temperature in the dark. Apoptosis was analyzed immediately using a flow cytometer (Beckman, USA).

Apoptosis in brain tissue was assessed in situ by TUNEL staining using a commercial kit (Beyotime, Cat#C1089) on frozen sections. Briefly, frozen sections were equilibrated to room temperature and fixed in 4 % paraformaldehyde for 30 min. After permeabilization with 0.1 % Triton X-100 in PBS for 5 min at room temperature, sections were washed and incubated with the TUNEL reaction mixture for 1 h at 37 °C in a dark, humidified chamber. After incubation, sections were washed three times with PBS. Cell nuclei were counterstained with DAPI. Stained sections were mounted with anti-fade mounting medium and visualized using a confocal laser-scanning microscope. The number of TUNEL-positive cells in the substantia nigra was quantified from multiple random fields per sample using ImageJ software.

### 2.12 Seahorse XF cell mito stress test

Real-time mitochondrial respiration was measured using a Seahorse XF96 Extracellular Flux Analyzer with the XF Cell Mito Stress Test Kit (Agilent Technologies, Cat #103015–100). To profile mitochondrial function, cells were treated sequentially with oligomycin (1.5 μM), the uncoupler FCCP (1 μM), and rotenone plus antimycin A (0.5 μM). The oxygen consumption rate (OCR) was recorded throughout. Final OCR values were normalized to the well's protein concentration using BCA method.

### 2.13 Detection of reactive oxygen species (ROS)

Mitochondrial superoxide levels in SH-SY5Y cells were measured using MitoSOX Red (Beyotime, Cat#S0061S). After treatment, cells were incubated with 5 μM MitoSOX Red at 37 °C for 40 min in the dark. Following staining, cells were washed twice with warm PBS, gently trypsinized, and resuspended in PBS for analysis. Fluorescence intensity was immediately measured by flow cytometry.

Superoxide levels in the substantia nigra were assessed using dihydroethidium (DHE; Beyotime, Cat#S0063). Frozen brain sections were fixed with acetone for 30 min at room temperature and then incubated with 5 μM DHE for 30 min at 37 °C in the dark. After DHE staining, sections were washed with PBS, and nuclei were counterstained with DAPI. Images were captured under consistent exposure settings using a confocal laser-scanning microscope. Fluorescence intensity was quantified from multiple random fields per sample using ImageJ software.

### 2.14 Mitochondrial membrane potential measurements and mitotracker staining

Mitochondrial membrane potential was assessed in SH-SY5Y cells using the JC-1 assay kit (Beyotime, Cat#C2003S). Cells were plated on glass coverslips in 24-well plates. After treatments, the culture medium was replaced with JC-1 staining solution and incubated at 37 °C for 20 min. After two washes with assay buffer, cells were immediately visualized under a fluorescence microscope. The red/green fluorescence intensity ratio (JC-1 aggregates/monomers, representing high/low mitochondrial membrane potential) was quantified from multiple cells per condition using ImageJ software to evaluate mitochondrial membrane potential.

Mitochondrial membrane potential and mitochondrial morphology in SH-SY5Y cells were further examined using MitoTracker Red CMXRos (Beyotime, Cat#C1035). For flow cytometric analysis of relative MMP, cells were stained with 200 nM MitoTracker Red for 30 min at 37 °C, harvested, and mean fluorescence intensity was immediately measured. For assessment of mitochondrial morphology, cells grown on glass coverslips were stained, fixed in 4 % paraformaldehyde, and imaged by confocal microscopy.

### 2.15 Nucleocytoplasmic separation

Nucleocytoplasmic separation was performed using a cell nucleocytoplasmic separation kit (Abbkine, Cat#KTP3002). SH-SY5Y cells (2 × 10^6^) were collected by centrifugation at 500×*g* for 5 min. The pellet was resuspended in ice-cold PBS and centrifuged again at 500×*g* for 2–3 min. After discarding the supernatant, 200 μL of ice-cold cytoplasmic solution A was added to the pellet and vortexed vigorously for 5 s to fully resuspend the cells. The suspension was incubated on ice for 15 min to induce cell swelling. Then, 10 μL of ice-cold cytoplasmic solution B was added, followed by vortexing at maximum speed for 5 s and incubation on ice for 1–2 min. The mixture was centrifuged at 16,000×*g* for 5 min at 4 °C. The supernatant (cytoplasmic fraction) was transferred to a clean pre-chilled tube and kept on ice. The pellet was resuspended in 100 μL of ice-cold nuclear extraction solution and maintained on ice. Over a 30-min period, the sample was vortexed every 3–5 min for 15 s, avoiding foam formation. Finally, the suspension was centrifuged at 16,000×*g* for 15 min at 4 °C. The resulting nuclear supernatant was aliquoted into pre-cooled tubes and stored at −80 °C.

### 2.16 Mitochondrial isolation

Mitochondrial and cytosolic fractions were prepared using a commercial mitochondria isolation kit (Beyotime, Cat#C3601) through differential centrifugation. After treatment, cells were collected by centrifugation at 200×*g* for 10 min at 4 °C and washed with PBS. The pellet was resuspended in 1 mL of ice-cold mitochondrial isolation reagent, incubated on ice for 15 min, and homogenized with 35 strokes in a pre-chilled glass Dounce homogenizer. Complete cell disruption was verified by trypan blue staining. The homogenate was first centrifuged at 600×*g* for 10 min at 4 °C to remove nuclei and unbroken cells. The supernatant was transferred to a new tube and centrifuged at 11,000×*g* for 10 min at 4 °C to obtain the mitochondrial fraction. The resulting supernatant, representing the cytosolic fraction (mitochondria-depleted), was collected and further clarified by centrifugation at 12,000×*g* for 10 min. The mitochondrial pellet was washed and resuspended in appropriate lysis buffer. Protein concentrations of both mitochondrial and cytosolic fractions were determined using a BCA assay.

### 2.17 Triton X-114 phase partitioning

Hydrophobic proteins were isolated using Triton X-114 phase separation. SH-SY5Y cells, treated with or without 1.5 mM MPP^+^ for 24 h, were lysed on ice for 30 min in a buffer consisting of 10 mM Tris-HCl (pH 7.4), 150 mM NaCl, 1 % (v/v) Triton X-114, and protease inhibitor cocktail [26]. Lysates were clarified by centrifugation at 15,000×*g* for 30 min at 4 °C. The supernatant was incubated at 37 °C for 3–5 min until turbidity appeared, followed by centrifugation at 400×*g* for 4 min at room temperature [27]. The lower detergent phase, enriched in hydrophobic proteins, was carefully collected. The detergent was diluted, and protein concentration was determined by Bradford assay for subsequent Western blot analysis.

### 2.18 Statistical analysis

Data are presented as mean ± SEM. The ‘n’ for in vitro studies represents the number of independent experiments; for in vivo studies, it indicates the number of animals per group. Statistical analyses were conducted using GraphPad Prism software. Normality of data distribution was evaluated with the Shapiro–Wilk test. For comparisons between two groups, an unpaired two-tailed Student's t-test was used. For comparisons among three or more groups, one-way analysis of variance (ANOVA) was employed, followed by Tukey's post hoc test for multiple comparisons. A p-value below 0.05 was considered statistically significant. Specific significance levels in the figures are denoted as ∗p < 0.05, ∗∗p < 0.01, and ∗∗∗p < 0.001.

## 3 Results

### 3.1 GBP2 expression is upregulated in PD patients, MPTP-treated/A53T mice and MPP^+^-induced/A53T α-synuclein-overexpressing SH-SY5Y cells

**Fig. 1:**
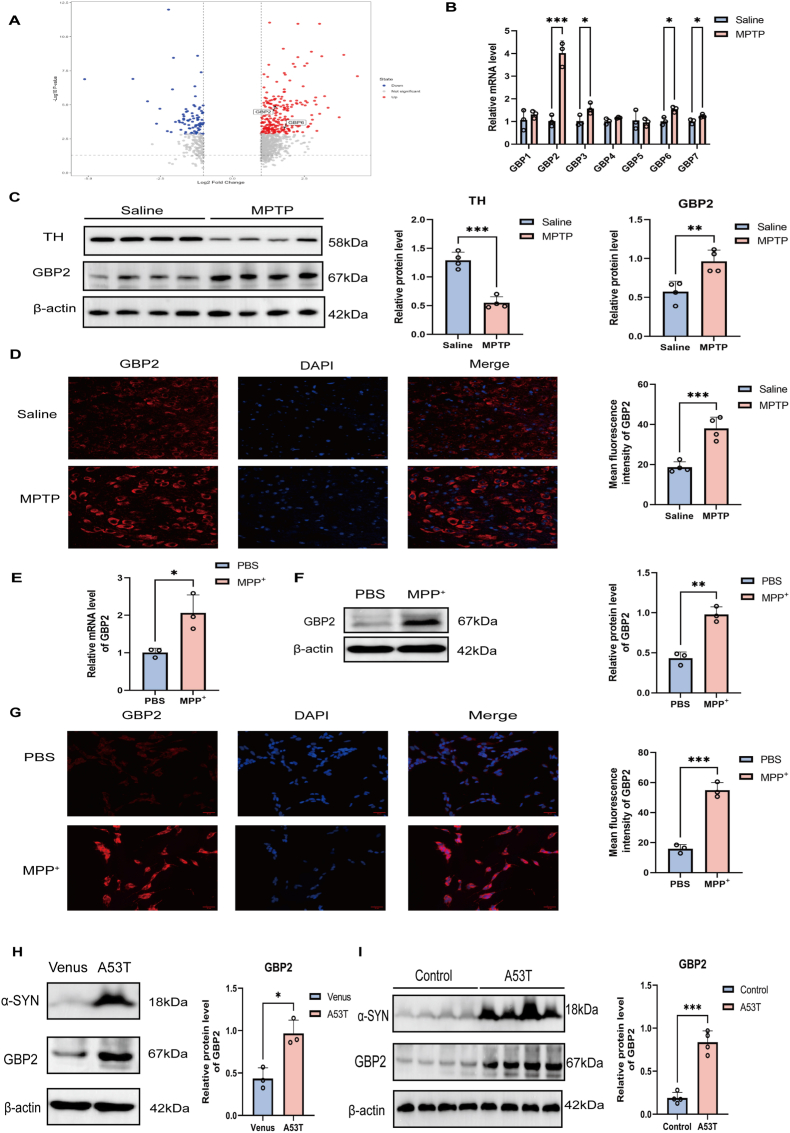
GBP2 expression is significantly upregulated in PD models. (A) Bioinformatic analysis of transcriptomic data from the GEO database (GSE106608) showing mRNA expression levels of GBP family members in the substantia nigra of PD patients and healthy controls. (B) RT-qPCR analysis of GBP mRNA levels in the substantia nigra of MPTP-induced PD mice and control mice (n = 3 mice per group). (C) Representative western blots and quantification of TH and GBP2 protein levels in the substantia nigra of the indicated groups (n = 4 mice per group). (D) Representative immunofluorescence images showing GBP2 expression in the substantia nigra (n = 4 mice per group). Scale bar, 20 μm. (E–G) SH-SY5Y cells were treated with or without 1.5 mM MPP^+^ for 24 h (n = 3 independent biological replicates). (E) RT-qPCR, (F) Western blot, and (G) immunofluorescence staining showing the upregulation of GBP2 in MPP^+^ treated SH-SY5Y cells. Scale bar, 50 μm. Data are presented as mean ± SEM. (H) Representative Western blot showing GBP2 protein levels in SH-SY5Y cells after lentivirus-mediated A53T α-synuclein-overexpressing (n = 3 independent biological replicates). (I) Representative Western blot showing GBP2 protein levels in the substantia nigra of 13-month-old A53T transgenic mice and their wild-type littermates (n = 4 mice per group). ∗p < 0.05, ∗∗p < 0.01, ∗∗∗p < 0.001 for comparisons between the indicated groups.

Bioinformatic analysis of a public transcriptomic dataset (GEO) revealed significant upregulation of *GBP2* and *GBP6* in the substantia nigra of PD patients compared with healthy controls (n = 8 patients per group) (Fig. 1A). To experimentally validate this finding, we utilized an MPTP-induced mouse model of PD. The successful induction of the PD model was confirmed by significant impairments in behavioral tests and a marked reduction in TH-positive neurons in the substantia nigra (Fig. S1A–B). RT-qPCR analysis further verified the upregulation of *GBP2*, *GBP3*, *GBP6*, and *GBP7* in the substantia nigra of PD mice, with *GBP2* showing the most marked upregulation (4-fold increase, *P* < 0.001, n = 3 mice per group) compared with the control group (Fig. 1B). Given that GBP2 showed the most prominent upregulation, we focused subsequent investigations on its functional role in PD. Western blot analysis corroborated the upregulation of GBP2 in PD mice and revealed an expression pattern inversely correlated with that of TH, a marker of dopaminergic neurons in the substantia nigra (Fig. 1C). In addition, immunofluorescence staining showed that GBP2 expression was predominantly upregulated in the substantia nigra of PD mice compared with controls (Fig. 1D). To establish an in vitro PD model, the optimal concentration of MPP^+^ was determined using a CCK-8 assay; based on the results, a treatment regimen of 1.5 mM MPP^+^ for 24 h was selected for subsequent experiments (Fig. S1C). Consistent with the in vivo data, GBP2 expression was also significantly elevated in SH-SY5Y cells treated with 1.5 mM MPP^+^ for 24 h, as assessed by RT-qPCR, Western blot, and immunofluorescence staining (Fig. 1E–G). To determine whether GBP2 upregulation is specifically associated with PD pathology rather than merely an acute stress response to MPTP/MPP^+^, we employed two chronic models: A53T transgenic mice and SH-SY5Y cells with lentivirus-mediated overexpression of A53T α-synuclein. Results confirmed that GBP2 levels were significantly elevated compared to controls in both chronic settings (Fig. 1H and I). In summary, these results demonstrate that GBP2 expression is elevated across multiple PD models, indicating a role for GBP2 in PD pathogenesis.

### 3.2 GBP2 knockdown protects against MPP^+^/MPTP-induced neurotoxicity in vitro and in vivo

**Fig. 2:**
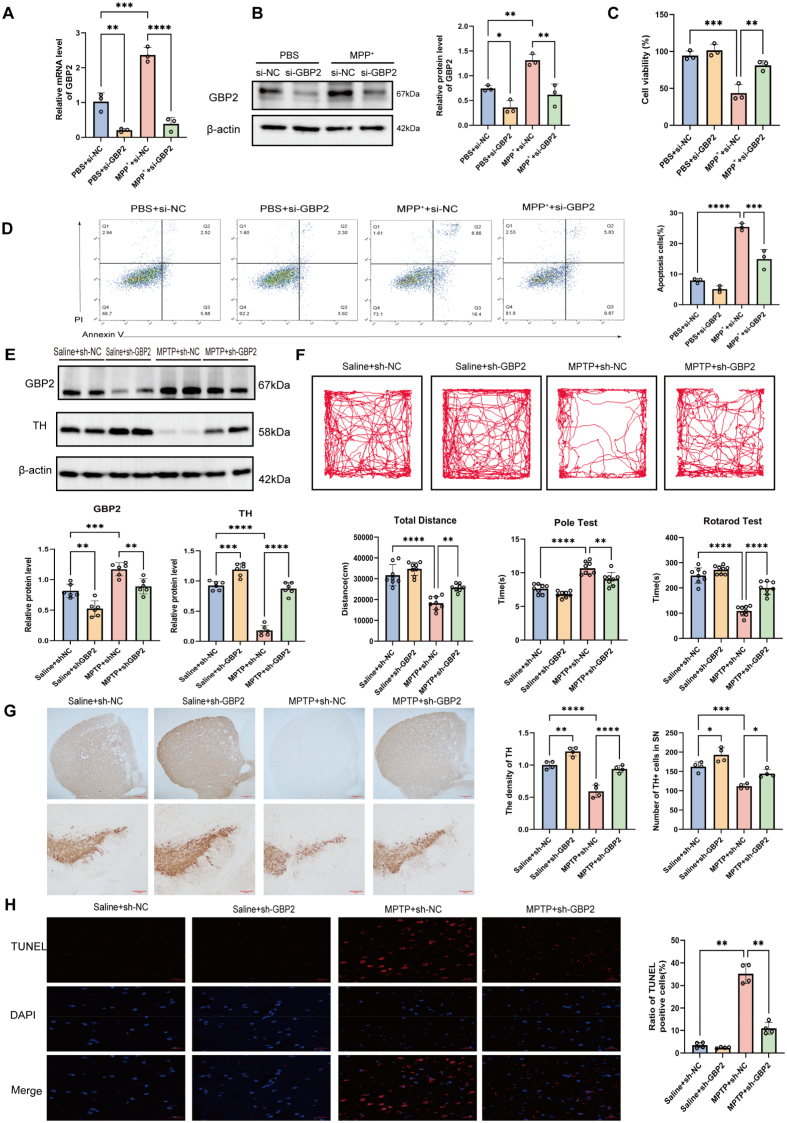
Genetic knockdown of GBP2 attenuates apoptosis and ameliorates motor deficits in PD models. (A, B) SH-SY5Y cells were transfected with negative control siRNA (si-NC) or GBP2-specific siRNA (si-GBP2) and then treated with or without MPP^+^ (n = 3 independent biological replicates). (A) RT-qPCR and (B) Western blot analysis validating the efficiency of GBP2 knockdown. (C) CCK-8 assay showing that GBP2 knockdown significantly attenuated MPP^+^-induced loss of cell viability (n = 3 independent biological replicates). (D) Flow cytometric analysis of apoptosis by Annexin V/PI staining, demonstrating that GBP2 knockdown reduced MPP^+^-induced apoptosis (n = 3 independent biological replicates). (E) Western blot analysis of GBP2 and TH protein levels in the substantia nigra of mice injected with AAV9-control (sh-NC) or AAV9-shGBP2 (sh-GBP2) and treated with MPTP or saline. GBP2 knockdown mitigated the MPTP-induced loss of TH protein (n = 6 mice per group). (F) Motor behavioral tests (open field, pole, and rotarod) showing that GBP2 knockdown ameliorated MPTP-induced motor deficits (n = 8 mice per group). (G) Representative IHC staining of the substantia nigra and striatum, showing ameliorated neuronal loss and pathological morphology in sh-GBP2 mice after MPTP treatment (n = 4 mice per group). Scale bar, 50 μm. (H) Representative TUNEL staining (red) in the substantia nigra, with DAPI (blue) counterstain for nuclei. GBP2 knockdown reduced MPTP-induced apoptosis (n = 4 mice per group). Scale bar, 20 μm. Data are presented as mean ± SEM. ∗p < 0.05, ∗∗p < 0.01, ∗∗∗p < 0.001 for comparisons between the indicated groups.

To investigate the role of GBP2, we used RNA interference in SH-SY5Y cells exposed to MPP^+^. We transfected cells with GBP2-specific siRNA (si-GBP2) or negative control siRNA (si-NC) and confirmed the knockdown efficiency (Fig. 2A–B). To evaluate the functional impact of GBP2 knockdown, we first performed CCK-8 assays, which showed that under MPP^+^ treatment, cells transfected with si-GBP2 maintained significantly higher viability than those transfected with si-NC (Fig. 2C). Consistently, flow cytometric analysis revealed a marked reduction in apoptosis in the si-GBP2 group compared with the si-NC group upon MPP^+^ exposure (Fig. 2D). Next, to examine the role of GBP2 in vivo, we induced GBP2 knockdown (sh-GBP2) in the mouse substantia nigra via AAV9-mediated delivery and verified the knockdown efficiency (n = 4 mice per group) (Fig. 2E). Consistent with the in vitro findings, GBP2 knockdown alleviated MPTP-induced motor deficits, as evidenced by behavioral tests and preserved TH expression (Fig. 2E–F). Additionally, compared to the MPTP + sh-NC group, the MPTP + sh-GBP2 group exhibited a 30 % higher count of TH^+^ neurons in the substantia nigra (*P <* 0.05) and a 50 % higher striatal TH density (*P <* 0.0001) (Fig. 2G). Moreover, TUNEL staining showed a significant decrease in apoptotic cells in sh-GBP2 mice compared with controls under MPTP treatment (Fig. 2H). Together, these findings demonstrate that GBP2 promotes neuronal apoptosis in the substantia nigra and aggravates MPTP-induced behavioral deficits, highlighting its pathogenic role in PD progression.

### 3.3 GBP2 accumulates in mitochondria and exacerbates mitochondrial dysfunction in MPP^+^/MPTP-induced PD models

**Fig. 3:**
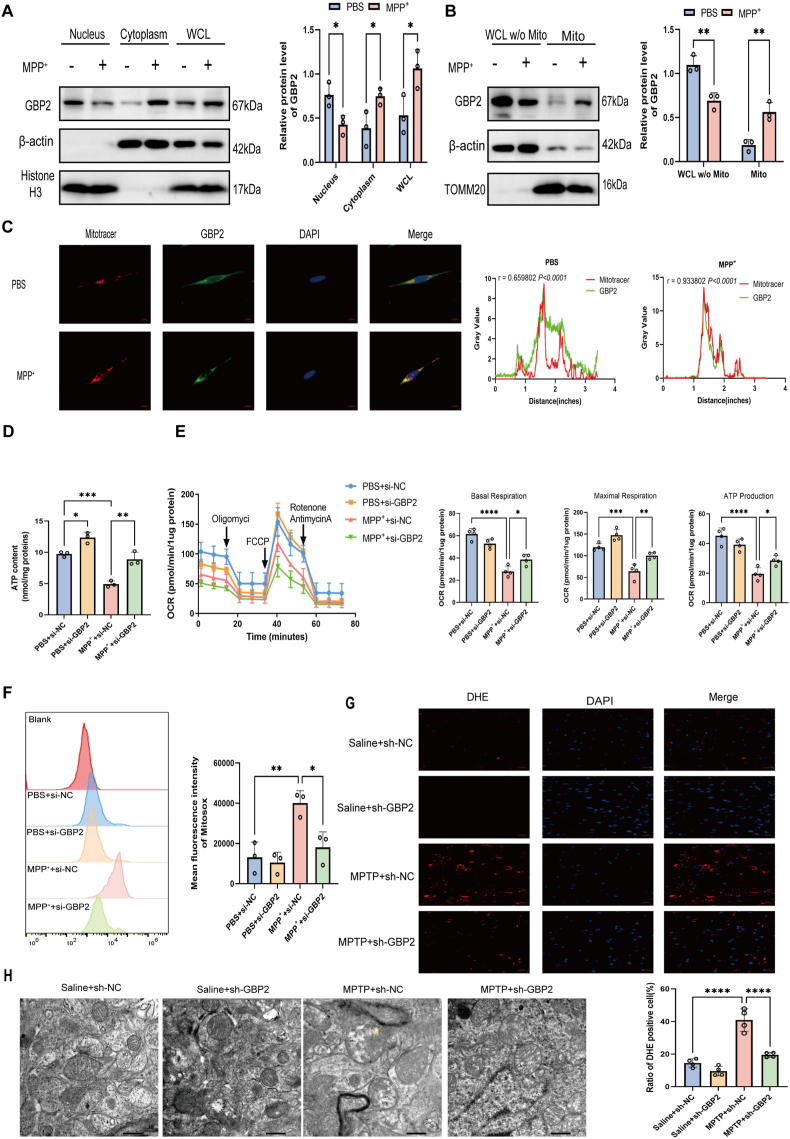
GBP2 accumulates in mitochondria and its knockdown ameliorates mitochondrial dysfunction and structural damage (A) Nucleocytoplasmic fractionation of SH-SY5Y cells treated with or without MPP^+^. β-actin and Histone H3 serve as cytoplasmic and nuclear markers, respectively (n = 3 independent biological replicates). (B) Mitochondrial and cytosolic fractions from cells treated with or without MPP^+^ were analyzed by western blotting for GBP2. TOMM20 and β-actin serve as mitochondrial and cytosolic markers, respectively (n = 3 independent biological replicates). (C) Immunofluorescence confocal microscopy showing colocalization of GBP2 (green) with a Mitotracker (red) in SH-SY5Y cells treated with or without MPP^+^ (n = 3 independent biological replicates). Scale bar, 10 μm. (D–F) SH-SY5Y cells transfected with si-NC or si-GBP2 were treated with MPP^+^ (n = 3 independent biological replicates). (D) Cellular ATP levels, (E) quantification of mitochondrial respiration capacity, and (F) mitochondrial ROS levels (MitoSOX Red staining) were measured. GBP2 knockdown significantly ameliorated MPP^+^-induced mitochondrial dysfunction. (G) Representative DHE staining (red) for superoxide in the substantia nigra of mice from the indicated groups (n = 4 mice per group). Scale bar, 20 μm. (H) Representative transmission electron microscopy (TEM) images of mitochondria in the substantia nigra. Yellow arrows indicate damaged cristae; white arrows indicate swollen mitochondria. Scale bar, 500 nm. Data are presented as mean ± SEM. ∗p < 0.05, ∗∗p < 0.01, ∗∗∗p < 0.001 for comparisons between the indicated groups.

To explore the mechanism by which GBP2 contributes to PD, we first examined its subcellular localization upon MPP^+^ treatment. Nucleocytoplasmic fractionation showed an increase in cytoplasmic GBP2 in SH-SY5Y cells after MPP^+^ exposure (Fig. 3A). To further determine the precise subcellular distribution of GBP2, we isolated mitochondrial fractions and performed Western blot analysis, which revealed a significant increase in mitochondrial GBP2 following MPP^+^ stimulation (Fig. 3B). Immunofluorescence staining corroborated these findings, showing increased colocalization of GBP2 with mitochondria in MPP^+^-treated SH-SY5Y cells (Fig. 3C). Together, these results indicate that GBP2 exerts its functional effects under MPP^+^ stress through mitochondrial targeting.

We next assessed mitochondrial changes resulting from GBP2 knockdown. Under MPP^+^ treatment, the ATP level in the si-GBP2 group was elevated by 1.8-fold relative to the si-NC group (n = 3 independent biological replicates, *P* < 0.01) (Fig. 3D). To determine whether GBP2 regulates mitochondrial function, we analyzed mitochondrial respiration via Seahorse XF assay. Knockdown of GBP2 effectively enhanced the oxygen consumption rate (OCR) in MPP^+^-treated SH-SY5Y cells (Fig. 3E), demonstrating that reducing GBP2 levels improves oxidative phosphorylation capacity under PD-stress. Moreover, GBP2 knockdown enhanced mitochondrial membrane potential (assessed by JC-1 staining, Fig. S2) and reduced mitochondrial ROS levels (assessed by MitoSOX staining, Fig. 3F) in MPP^+^-treated SH-SY5Y cells. Consistent with the in vitro data, DHE staining revealed a 50 % reduction in ROS levels in the substantia nigra of sh-GBP2 mice (n = 4 mice per group, *P* < 0.0001) (Fig. 3G). Transmission electron microscopy (TEM) was used to evaluate mitochondrial ultrastructure, demonstrating that sh-GBP2 mice exhibited significantly less mitochondrial swelling, reduced disruption of inner and outer membranes, and preserved cristae integrity compared to the sh-NC group (Fig. 3H).

In summary, our findings indicate that under PD-related stress, GBP2 accumulates in mitochondria and acts as a key driver of mitochondrial dysfunction. Knockdown of GBP2 effectively sustains mitochondrial bioenergetics, attenuates oxidative stress, and protects against ultrastructural damage, thereby establishing a direct role for GBP2 in mitochondrial pathogenesis in PD.

### 3.4 GBP2 knockdown activates NIX-mediated mitophagy to alleviate PD damage

**Fig. 4:**
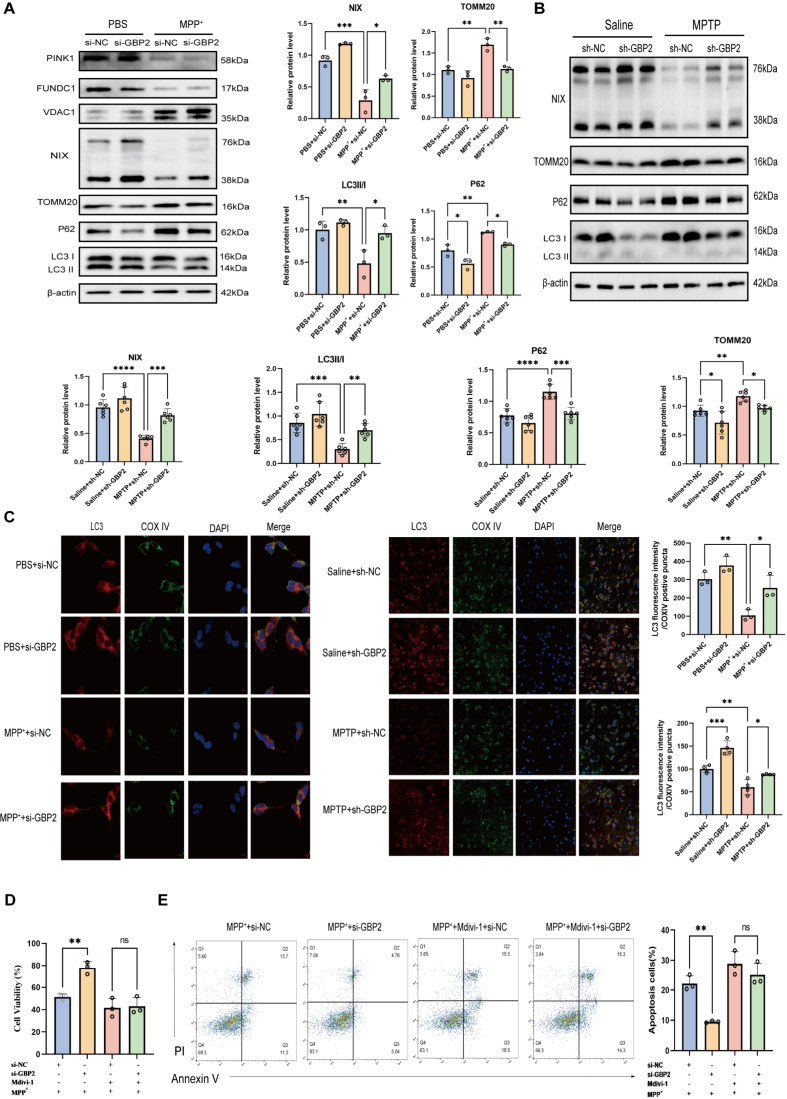
GBP2 knockdown enhances mitophagy. (A) Western blot analysis of key mitophagy markers in SH-SY5Y cells transfected with si-NC or si-GBP2 and treated with or without MPP^+^ (n = 3 independent biological replicates). GBP2 knockdown specifically upregulated NIX and the LC3-II/LC3-I ratio, while downregulating p62 and TOMM20. (B) Western blot analysis of the indicated proteins in the substantia nigra of mice from the indicated groups. GBP2 knockdown enhanced NIX expression and the LC3-II/LC3-I ratio, while reducing p62 and TOMM20 levels under MPTP treatment (n = 6 mice per group). (C) Representative immunofluorescence images showing colocalization of LC3 (red) with the mitochondrial marker COX-IV (green) in (Left) SH-SY5Y cells (n = 3 independent biological replicates) and (Right) the substantia nigra (n = 4 mice per group) of mice under the indicated conditions. Scale bars, 10 μm (cells); 20 μm (tissue). (D, E) SH-SY5Y cells transfected with si-NC or si-GBP2 were pretreated with the mitophagy inhibitor Mdivi-1 (10 μM) or DMSO for 6 h prior to MPP^+^ exposure (n = 3 independent biological replicates). (D) CCK-8 assay for cell viability and (E) flow cytometry for apoptosis showed that Mdivi-1 treatment partially reversed the protective effects of GBP2 knockdown (n = 3 independent biological replicates). Data are presented as mean ± SEM. ∗p < 0.05, ∗∗p < 0.01, ∗∗∗p < 0.001 for comparisons between the indicated groups.

We next examined the effect of GBP2 on mitophagy in SH-SY5Y cells. Under MPP^+^ stress, GBP2 knockdown specifically increased protein levels of NIX and the LC3-II/LC3-I ratio, while decreasing p62 and the mitochondrial outer membrane protein TOMM20. In contrast, the expression of other mitophagy-related proteins, including PINK1, VDAC1, and FUNDC1, remained unaltered (Fig. 4A). Consistent with the in vitro results, Western blot analysis showed that under MPTP treatment, the substantia nigra of sh-GBP2 mice exhibited upregulation of NIX and the LC3-II/LC3-I ratio, along with downregulation of p62 and TOMM20, compared with sh-NC controls (Fig. 4B). Similarly, immunofluorescence colocalization analysis of LC3 with the mitochondrial marker COX-IV, a well-established indicator of mitophagy, revealed a significant increase in the si-GBP2 group relative to the si-NC group. Enhanced LC3 and COX-IV colocalization was also observed in the substantia nigra of sh-GBP2 mice compared with sh-NC controls following MPTP administration (Fig. 4C). To determine whether mitophagy flux is required for the protective effect of GBP2 knockdown, we pretreated SH-SY5Y cells with the mitophagy inhibitor Mdivi-1. Mdivi-1 treatment largely reversed the anti-apoptotic effect and improved cell viability resulting from GBP2 knockdown, as demonstrated by CCK-8 assay and flow cytometry (Fig. 4D and E). Collectively, these findings strongly indicate that GBP2 knockdown protects against MPTP/MPP^+^-induced neuronal damage by enhancing mitophagy, likely by regulating NIX protein stability.

### 3.5 GBP2 directly interacts with NIX via its GTPase domain to modulate NIX stability

**Fig. 5:**
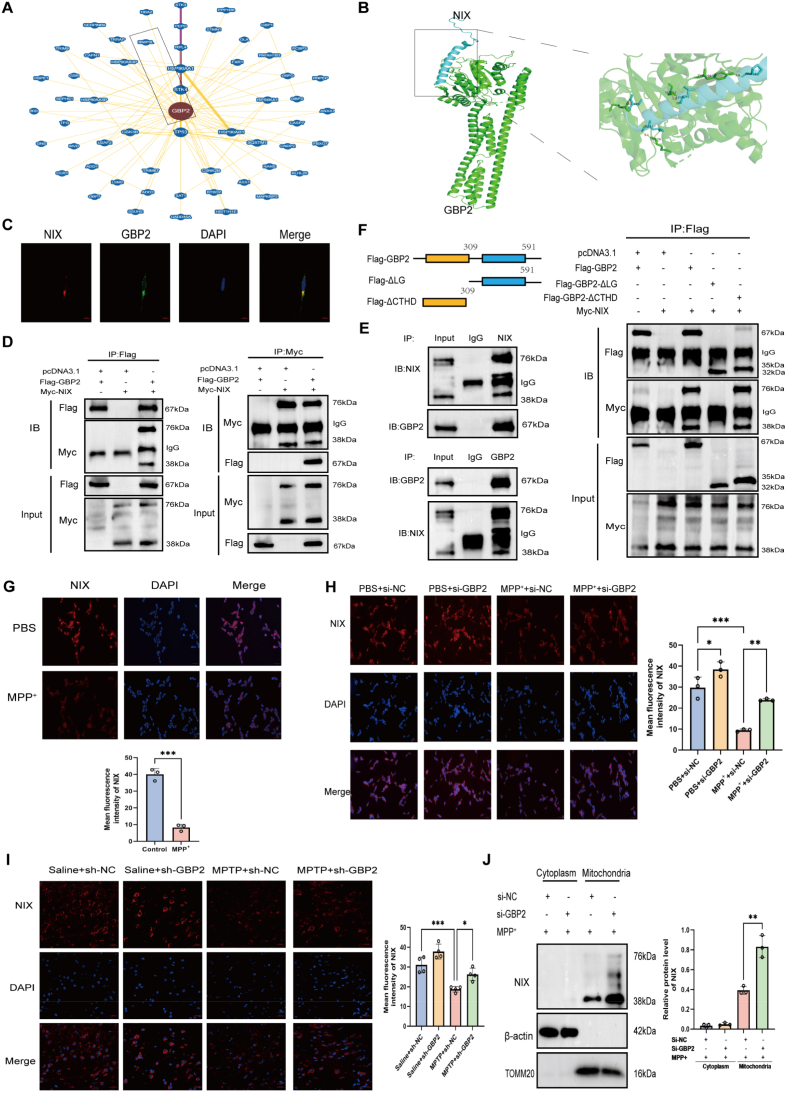
GBP2 directly interacts with NIX via its GTPase domain and facilitates its degradation. (A) Prediction of GBP2-interacting proteins from the BioGRID database, identifying NIX as a candidate. (B) ZDOCK prediction of the three-dimensional structural interaction between GBP2 and NIX, revealing a high-confidence binding interface. (C) Immunofluorescence confocal microscopy showing colocalization of endogenous GBP2 (green) and NIX (red) in SH-SY5Y cells. Scale bar, 10 μm. (D) Co-immunoprecipitation (Co-IP) assays in HEK293T cells overexpressing Flag-GBP2 and Myc-NIX, confirming their interaction. (E) Endogenous Co-IP validating the interaction between GBP2 and NIX in SH-SY5Y cells. (F) Co-IP assays in HEK293T cells expressing Myc-NIX and full-length Flag-GBP2, GTPase domain-deleted GBP2 (ΔLG), or C-terminal helical domain-deleted GBP2 (ΔCTHD). The interaction is dependent on the LG domain. (G–J) GBP2 knockdown rescues NIX protein expression. (G) MPP^+^ treatment reduces NIX protein levels in SH-SY5Y cells, as shown by immunofluorescence (n = 3 independent biological replicates). Scale bar, 50 μm. (H, I) Immunofluorescence analysis of NIX in (H) MPP^+^-treated SH-SY5Y cells (n = 3 independent biological replicates) and (I) the substantia nigra of MPTP-treated mice (n = 4 mice per group) demonstrates that GBP2 knockdown prevents NIX downregulation. Scale bars, 50 μm (cell) and 20 μm (tissue) (J) Western blot of NIX in mitochondrial fractions from SH-SY5Y cells confirms the increase in NIX upon GBP2 knockdown (n = 3 independent biological replicates). Data are presented as mean ± SEM. ∗p < 0.05, ∗∗p < 0.01, ∗∗∗p < 0.001 for comparisons between the indicated groups.

To explore the mechanism by which GBP2 regulates mitophagy in SH-SY5Y cells, we first queried the BioGRID database to identify potential GBP2-interacting proteins. Among these, the mitophagy receptor NIX was selected for further validation based on its relevance to our phenotype (Fig. 5A). ZDOCK analysis predicted a high-confidence three-dimensional interaction between GBP2 and NIX (Fig. 5B), suggesting a binding interface involving the LG domain of GBP2 (residues 1, 52, 76, 244) and the transmembrane (TM) domain (Table S1 shows the domains of NIX) and adjacent regions of NIX (residues 167, 179, 187, 190) (Fig. 5B). Immunofluorescence staining confirmed the co-localization of GBP2 with NIX in both cellular and animal tissue contexts (Fig. 5C and S3). To validate this interaction, Co-IP assays were performed in HEK293T cells expressing exogenous GBP2 and NIX. As shown in Fig. 5D, ectopically expressed GBP2 co-precipitated with NIX, and reciprocally, NIX co-precipitated with GBP2. Furthermore, endogenous Co-IP assays in SH-SY5Y cells confirmed a robust interaction between GBP2 and NIX at physiological expression levels (Fig. 5E). To map the specific domain of GBP2 responsible for interacting with NIX, we generated two truncated variants: a Flag-tagged GBP2 lacking the large GTPase domain (GBP2-ΔLG) and a Flag-tagged GBP2 lacking the C-terminal helical domain (GBP2-ΔCTHD). These constructs were expressed in HEK293T cells and subjected to Co-IP assays. Results showed that deletion of the LG domain completely abolished GBP2–NIX binding, whereas the GBP2-ΔCTHD variant retained the ability to interact with NIX (Fig. 5F). Together, these data demonstrate that GBP2 directly binds to NIX and that this interaction depends on its LG domain.

Given the direct GBP2–NIX interaction, we hypothesized that GBP2 upregulation might regulate NIX protein stability. NIX expression was significantly decreased in both MPP^+^-treated SH-SY5Y cells and A53T transgenic mice (Fig. 5G and S4). To test whether GBP2 mediates this downregulation, we knocked down GBP2 and observed a robust increase in NIX protein levels under both MPP^+^ (cellular model) and MPTP (animal model) treatments (Fig. 5H and I). Consistently, GBP2 knockdown also markedly elevated NIX levels in isolated mitochondrial fractions compared with controls (Fig. 5J). Collectively, these results define a novel pathway wherein GBP2, via direct interaction through its LG domain, targets NIX for destabilization.

### 3.6 GBP2 mediates NIX ubiquitination and degradation to suppress mitophagy

**Fig. 6:**
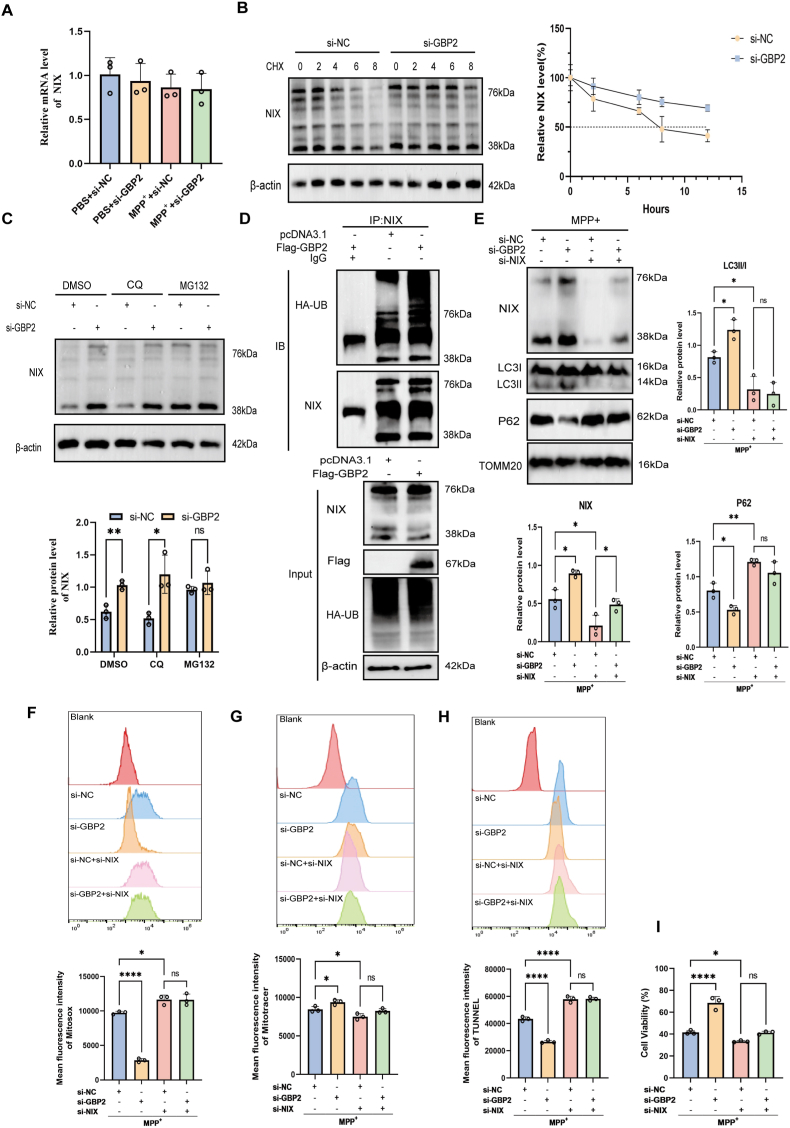
GBP2 promotes NIX ubiquitination and proteasomal degradation, and its protective effects on mitophagy and PD are NIX-dependent. (A) NIX mRNA levels in SH-SY5Y cells transfected with si-NC or si-GBP2 were measured by RT-qPCR. GBP2 knockdown did not alter NIX mRNA expression (n = 3 independent biological replicates). (B, C) SH-SY5Y cells transfected with si-NC or si-GBP2 were treated with MPP^+^. (B) Cells were treated with cycloheximide (CHX, 100 μg/ml) for the indicated times (n = 3 independent biological replicates). Western blot quantification shows that GBP2 knockdown prolonged the half-life of NIX protein. (C) Cells were treated with the proteasome inhibitor MG132 (10 μM) or the lysosome inhibitor chloroquine (CQ, 10 μM) for 6 h (n = 3 independent biological replicates). MG132, but not CQ, blocked the GBP2 knockdown-induced increase in NIX protein levels. (D) Ubiquitination assays in HEK293T cells co-expressing HA-Ub, with Flag-GBP2 or pcDNA3.1, showing that GBP2 overexpression enhances NIX ubiquitination. (E–I) SH-SY5Y cells were co-transfected with si-GBP2 and si-NIX (or respective controls) and treated with MPP^+^ (n = 3 independent biological replicates). NIX knockdown reversed the protective effects of GBP2 knockdown on: (E) mitophagy markers (LC3-II/I and p62) in mitochondrial fractions, (F) mitochondrial membrane potential, (G) mitochondrial ROS levels, (H) apoptosis, and (I) cell viability. Data are presented as mean ± SEM. ∗p < 0.05, ∗∗p < 0.01, ∗∗∗p < 0.001 for comparisons between the indicated groups.

We first asked whether GBP2 regulates NIX transcriptionally by measuring NIX mRNA levels via RT-qPCR. The results showed no significant difference in NIX mRNA levels between SH-SY5Y cells transfected with si-NC and those transfected with si-GBP2, indicating that GBP2 regulates NIX post-transcriptionally (Fig. 6A). To assess NIX protein stability, we performed cycloheximide (CHX) chase assays, which showed that GBP2 knockdown significantly extended the half-life of NIX protein in SH-SY5Y cells (Fig. 6B). These findings suggest that GBP2 promotes NIX degradation at the protein level. To identify the degradation pathway involved, SH-SY5Y cells transfected with si-NC or si-GBP2 were treated with chloroquine (CQ; a lysosomal inhibitor) or MG132 (a proteasomal inhibitor), and changes in NIX protein levels were assessed. The proteasome inhibitor MG132, but not the autophagy/lysosomal inhibitor chloroquine (CQ), blocked the increase in NIX protein levels induced by GBP2 silencing (Fig. 6C). Consistently, ectopic expression of GBP2 enhanced NIX ubiquitination in HEK293T cells (Fig. 6D). Together, these data demonstrate that GBP2 promotes NIX degradation via the ubiquitin–proteasome pathway.

To assess whether GBP2 regulates mitophagy in a NIX-dependent manner, we knocked down NIX under GBP2-knockdown conditions. NIX silencing largely reversed the enhancement of mitophagy caused by GBP2 knockdown in SH-SY5Y cells, as reflected by similar LC3-II/I ratios and p62 levels in mitochondrial fractions of the si-GBP2 and si-NC groups (Fig. 6E). Consistent with these observations, NIX knockdown abolished the differences in ROS and mitochondrial membrane potential levels between si-NC and si-GBP2 cells under MPP^+^ treatment (Fig. 6F and G). To determine whether NIX is required for the cytoprotective effects of GBP2 knockdown, we performed concurrent knockdown of both genes. The reduction in apoptosis and improvement in cell viability resulting from GBP2 knockdown were largely reversed by NIX silencing, as shown by flow cytometry and CCK-8 assays (Fig. 6H and I). In summary, these results establish NIX as the essential mediator through which GBP2 regulates mitophagy, mitochondrial function, and cell survival in PD models.

### 3.7 Geranylgeranylation is necessary for the mitochondrial accumulation and function of GBP2 and its inhibition confers therapeutic benefit

**Fig. 7:**
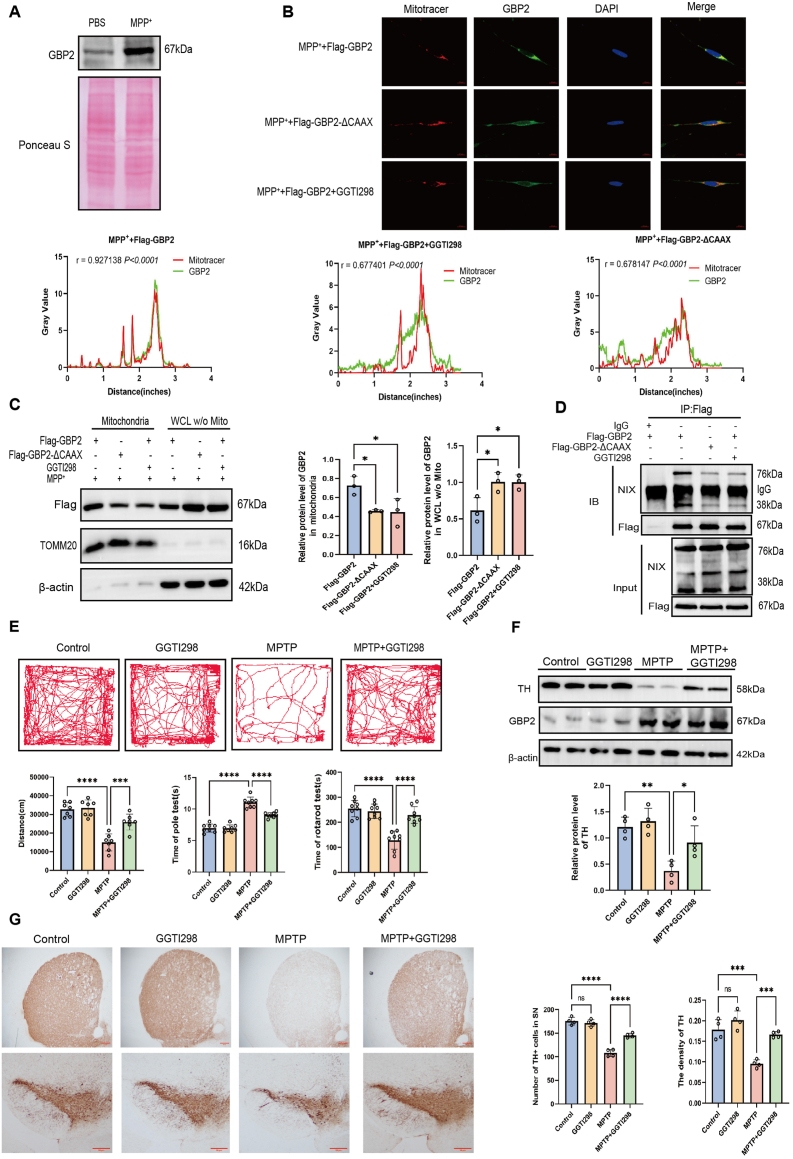
Geranylgeranylation is essential for GBP2 mitochondrial localization and function, and its inhibition protects against neurotoxicity. (A) Triton X-114 phase partitioning assay in SH-SY5Y cells treated with or without MPP^+^. MPP^+^ treatment shifted GBP2 toward the detergent phase, indicating increased hydrophobicity (n = 3 independent biological replicates). (B, C) SH-SY5Y cells were transfected with Flag-GBP2, Flag-GBP2-ΔCAAX, or treated with the geranylgeranylation inhibitor GGTI298 (10 μM) for 48 h (n = 3 independent biological replicates). (B) Immunofluorescence of GBP2 (green) and a Mitotracker (red) shows that disrupting geranylgeranylation reduces mitochondrial localization of GBP2. Scale bar, 10 μm. (C) Western blot of GBP2 in mitochondrial and cytosolic fractions confirms the reduction in mitochondrial targeting in Flag-GBP2-ΔCAAX group or GGTI298 group. (D) Co-IP assays in HEK293T cells show that GGTI298 treatment or expression of the GBP2-ΔCAAX mutant impairs the interaction between GBP2 and NIX. (E–G) MPTP-induced PD model mice were treated with GGTI298 or saline. (E) Behavioral tests (open field, pole, rotarod) demonstrate that GGTI298 treatment ameliorates MPTP-induced motor deficits (n = 8 mice per group). (F) Western blot and (G) representative IHC for TH in the substantia nigra and striatum show that GGTI298 treatment preserves dopaminergic neurons (n = 4 mice per group). Scale bar, 50 μm. Data are presented as mean ± SEM. ∗p < 0.05, ∗∗p < 0.01, ∗∗∗p < 0.001 for comparisons between the indicated groups.

To investigate whether post-translational modifications of GBP2 influence its role in PD, we performed Triton X-114 phase separation to assess its hydrophobicity under MPP^+^ treatment (Fig. 7A). Triton X-114 phase separation revealed an increased hydrophobic fraction of GBP2 upon MPP^+^ treatment, suggesting enhanced lipidation consistent with geranylgeranylation, as predicted from its CAAX motif [11]. To directly test the requirement for geranylgeranylation, we generated a GBP2 mutant lacking the CAAX prenylation motif (GBP2-ΔCAAX). Immunofluorescence and mitochondrial fractionation assays revealed that both pharmacological inhibition with GGTI298 and genetic ablation of the CAAX motif (GBP2-ΔCAAX) reduced the mitochondrial localization of GBP2, indicating that geranylgeranylation is required for its mitochondrial targeting (Fig. 7B and C). Furthermore, Co-IP experiments showed that GGTI298 treatment and GBP2-ΔCAAX expression each decreased the interaction between GBP2 and NIX, confirming that geranylgeranylation modulates GBP2–NIX binding (Fig. 7D). Together, these data indicate that MPP^+^ promotes esterification of GBP2, and specifically that geranylgeranylation is essential for its mitochondrial localization and subsequent interaction with NIX.

Based on these findings, we evaluated whether inhibiting geranylgeranylation could alleviate PD-related phenotypes. We administered GGTI298 to MPTP-induced PD model mice and found that the MPTP + GGTI298 group displayed significantly milder motor deficits than the MPTP-only group (Fig. 7E). Western blot analysis revealed that TH expression in the substantia nigra was 2.4-fold higher in the MPTP + GGTI298 group than in the MPTP group (n = 4 mice per group, P < 0.05; Fig. 7F). Consistently, IHC analysis revealed markedly higher TH levels in both the striatum and substantia nigra of GGTI298-treated mice (Fig. 7G). To determine whether GGTI298 exerts its protective effect via regulation of GBP2 geranylgeranylation, we treated cells with GGTI298 and MPP^+^ after transfecting them with Flag-GBP2-ΔCAAX, Flag-GBP2, or pcDNA3.1 (control). Cell viability in the MPP^+^ + GGTI298 + Flag-GBP2 group was significantly higher than in the MPP^+^ + GGTI298 + pcDNA3.1 group, whereas no significant change occurred in the Flag-GBP2-ΔCAAX group (Fig. S5A). Meanwhile, GGTI298 could also significantly alleviate the mitochondrial dysfunction induced by GBP2 overexpression (Fig.S5B). Together, these results demonstrate that pharmacological inhibition of geranylgeranylation by GGTI298 alleviates key parkinsonian phenotypes in vivo, significantly improving motor performance and preserving dopaminergic neurons.

## 4 Discussion

The pathogenesis of PD is closely associated with mitochondrial quality control, although the regulatory mechanisms, especially those involving alternative mitophagy pathways, remain incompletely elucidated [28]. In this context, our study identifies GBP2 as a critical and previously uncharacterized contributor to PD pathology. We delineate a mechanism whereby GBP2, which is upregulated under PD-relevant stress, is targeted to mitochondria via geranylgeranylation. Once there, GBP2 directly binds to the mitophagy receptor NIX and promotes its ubiquitin-mediated degradation. This cascade suppresses mitophagy, disrupts mitochondrial homeostasis, and ultimately drives dopaminergic neurodegeneration. Notably, pharmacological inhibition of GBP2 geranylgeranylation by GGTI298 effectively attenuates neurotoxicity in vivo. Our results not only establish GBP2 as a key mediator of PD pathogenesis but also reveal a non-canonical, GTPase-dependent mechanism for the precise regulation of receptor-mediated mitophagy, nominating GBP2 as a novel therapeutic target.

Historically, the GBP family has been primarily studied in the context of cell-autonomous immunity against intracellular pathogens [13,29]. In the context of PD, our findings reveal that GBP2 assumes a distinct pathogenic role. Its marked upregulation in the substantia nigra of PD models, coupled with the neuroprotective effects of its knockdown, establishes it as a key negative regulator of neuronal survival. Notably, mitophagy normally serves as a protective process and does not directly cause cell death [30,31]. However, when mitophagy becomes defective, it loses its protective function and instead allows cell death to proceed. This concept is clearly demonstrated in PD, where defective mitophagy worsens the progression of neurodegeneration [32]. Although prior work has shown that GBP2 suppresses breast cancer cell invasion by modulating Drp1-mediated mitochondrial fission, its potential influence on mitochondrial function and mitophagy remains unexplored [20]. In the present study, we demonstrate that GBP2 knockdown restored mitochondrial function and vitality of neurons, indicating GBP2 as an important pathological factor of PD.

Evidence suggests that NIX-mediated mitophagy serves as an important compensatory mechanism in PD [33]. Nix is a BH3-only BCL-2 family protein localized to the mitochondrial outer membrane. While its homodimerization is known to be essential for inducing mitophagy, the regulatory mechanisms controlling this process at the molecular level are still elusive [8]. In this study, we reveal that GBP2 is a key regulator of this pathway, acting by directly interacting with NIX to disrupt mitochondrial homeostasis. While the LG domain of GBP2 is known to mediate its antimicrobial functions [16,34], we find it is also critically required for its interaction with NIX. Deletion of this domain completely abolished GBP2–NIX binding, whereas the GBP2-ΔCTHD mutant retained interaction with NIX. Regarding the regulation mechanism of NIX, its cellular levels are tightly controlled by ubiquitination to avoid excessive mitochondrial clearance [35]. However, constitutive degradation of NIX risks untimely mitochondrial loss [36]. Here, we show that GBP2 directly interacts with NIX and promotes its ubiquitination and subsequent degradation via the ubiquitin–proteasome system. This results in a hyper-ubiquitination state of NIX, triggering a consequent decline in mitophagic flux. While the PINK1/Parkin pathway is a major genetic determinant of PD, its impairment often necessitates compensatory mechanisms [8]. Our finding that the GBP2-NIX axis is dysregulated across toxin-induced and genetic models suggests it represents a parallel and vulnerable pathway whose disruption contributes to pathology, particularly when canonical mitophagy is compromised. This delineates a specific pathogenic module beyond the core PINK1/Parkin machinery. Given that NIX acts as a compensatory mitophagy pathway, and its deficiency is not genetically determined, enhancing NIX-mediated mitophagy by GBP2 knockdown offers a potential therapeutic strategy for PD.

The discovery that geranylgeranylation acts as an upstream regulatory switch modulating the mitochondrial localization of GBP2 and its protein interactions significantly enhances the translational potential of our findings. Geranylgeranylation is a post-translational modification that attaches a geranylgeranyl group from geranylgeranyl pyrophosphate to a C-terminal cysteine, introducing a hydrophobic moiety that facilitates membrane association and proper subcellular localization [37]. In certain contexts, this modification also serves as a binding motif for interaction partners [38]. Structurally, the C terminus of the GTPase effector domain in GBP2 features a CAAX motif that targets it for geranylgeranylation. Previous studies reported that geranylgeranylation of GBP2 influences its ability to suppress viral propagation and motility [16,39].We therefore hypothesized that this lipid modification is critical for the role of GBP2 in exacerbating PD pathology [40]. Our data demonstrate that MPP^+^ induces a hydrophobic shift in GBP2. Additionally, the GBP2 ΔCAAX mutant exhibits reduced mitochondrial localization and a weaker interaction with NIX, consequently demonstrating that this lipid modification is essential for both its mitochondrial targeting and NIX binding. Most importantly, administration of GGTI298, a geranylgeranylation inhibitor, significantly alleviated MPTP-induced motor deficits and the loss of TH^+^ dopaminergic neurons (Fig. 7E–G), supporting the therapeutic potential of this pathway. This proof-of-concept study goes beyond mechanistic insight to propose a viable therapeutic strategy, suggesting that pharmacologically inhibiting GBP2 mitochondrial localization may represent a novel disease-modifying approach for PD.

Although our findings establish the GBP2–NIX–mitophagy axis, several key issues regarding its regulation and pathophysiological impact await further investigation. First, the pathogenic upregulation of GBP2 in PD is an area demanding deeper experimental investigation. Based on our literature review, we hypothesize that its upstream regulation may involve interferon-mediated responses, particularly through the JAK/STAT1 signaling axis and downstream transcription factors such as interferon regulatory factor 1 and interferon regulatory factor 7 [41]. Secondly, the E3 ligase mediating GBP2-dependent NIX degradation is still unidentified. A prime candidate is the SKP1-CUL1-F-box (SCF)– F-box and leucine-rich repeat protein 4 (FBXL4) complex (dependent on adaptor PPTC7), given its established role in mitochondrial NIX turnover [36,[42], [43], [44], [45]]. We propose that GBP2 likely acts as a scaffold/co-factor, possibly by regulating FBXL4 or PPTC7, to locally recruit or activate this degradation machinery. Thus, identifying this ligase and elucidating GBP2's exact role are vital future objectives. Moreover, our study demonstrates that the LG domain of GBP2 is involved in its interaction with NIX, while the precise amino acid residues involved remain uncharacterized, requiring further investigation. Crucially, although our study revealed the therapeutic potential of GGTI-298 for PD, its clinical translation requires further investigation. This includes assessing its correlation with clinical symptoms in patients and evaluating key pharmacological properties such as pharmacokinetics and blood-brain barrier penetration. Finally, mitophagy involves complex crosstalk among multiple pathways. While our study specifically focuses on the GBP2-NIX axis, its interaction with broader mitophagic networks remains to be explored and warrants further investigation.

Despite the insights gained, several limitations of this work merit consideration. Although geranylgeranylation of GBP2 is strongly inferred from indirect evidence (CAAX mutation, increased hydrophobicity, GGTI298 sensitivity; Fig. 7A–D), direct measurement was not feasible under our experimental conditions. Definitive confirmation would require an in vitro assay using [^3^H]-labeled geranylgeranyl pyrophosphate in future studies. Moreover, regarding therapeutic potential, the efficacy of GGTI298 in our model is promising; however, it should be noted that geranylgeranylation is a common modification across multiple proteins, including other small GTPases [46]. Additionally, primary neurons or neurons derived from induced pluripotent stem cells and human tissue should be incorporated to further strengthen the experimental results. Finally, direct neurochemical measurements (e.g., HPLC) would be valuable in future studies to definitively establish the biochemical correlates of the behavioral recovery and to strengthen the translational interpretation of the findings.

In summary, we have identified a novel and critical role for GBP2 in PD pathogenesis. The mechanistic cascade, encompassing its geranylgeranylation modulation and culminating in the suppression of NIX-mediated mitophagy, offers a new conceptual framework for understanding mitochondrial dysfunction in neurodegeneration. Ultimately, our findings nominate GBP2 and its associated pathway as promising targets for developing neuroprotective therapies for PD.

## Declaration of generative AI and AI-assisted technologies in the writing process

During the preparation of this work, the authors used Deepseek in order to correct grammatical errors. After using this tool, the authors reviewed and edited the content as needed and take full responsibility for the content of the publication.

## Funding

This work was supported by the 10.13039/501100001809Natural Science Foundation of China (82271275).

## CRediT authorship contribution statement

**Wenqi Cui:** Data curation, Formal analysis, Investigation, Methodology, Project administration, Software, Writing – original draft, Writing – review & editing. **Tianlu Wang:** Formal analysis, Methodology, Writing – review & editing. **Juan Feng:** Funding acquisition, Resources, Writing – review & editing.

## Declaration of competing interest

The authors declare that they have no known competing financial interests or personal relationships that could have appeared to influence the work reported in this paper.
