# Supplementary material for: Upregulated GBP2 exacerbates Parkinson's disease pathogenesis by impairing NIX-dependent mitophagy
Source: Redox Biol. 2026 Jan 17;90:104029. doi: 10.1016/j.redox.2026.104029 (PMC12859793; doi:10.1016/j.redox.2026.104029)
Supplement: Multimedia component 1 [file mmc1.docx]

Table S1. Domains of GBP2s

| Protein | Domains | Containing domains |
| --- | --- | --- |
|  |  |  |
| GBP2 | N-terminal globular large GTPase domain(LG) |  |
|  | C‑terminal helical domain  (CTHD) | Hinge; Middle domain (MD) and GTPase effector domain (GED) |
|  |  |  |
| NIX | LC3-interacting region (LIR) |  |
|  | Minimal essential region (MER) |  |
|  | BCL2 homology domain 3(BH3) |  |
|  | Transmembrane domain (TM) |  |

Table S2. Sequences of si-RNA

| Gene (human) | Sequence (5’- 3’) |
| --- | --- |
| si-GBP2 | F: CCAAAUGUUCCAGAGGAAATT  R: UUUCCUCUGGAACAUUUGGTT |
| si-NIX | F: GUCAGAAGAAGAAGUUGUA  R: UACAACUUCUUCUUCUGAC |
| si-NC | F: UUCUCCGAACGUGUCACGUTT  R: ACGUGACACGUUCGGAGAATT |

Table S3. Sequences of primers

| Gene | Sequence (5’- 3’) |
| --- | --- |
| h-GBP2 | F: TGCCCACTATGAACAGCAGAT  R: GTCATCTCGCCTTGCTTCCA |
| h-GAPDH | F: GCACCGTCAAGGCTGAGAAC  R: TGGTGAAGACGCCAGTGGA |
| h-NIX | F: ATGTCGTCCCACCTAGTCGAG  R: TGAGGATGGTACGTGTTCCAG |
| m-GBP1 | F: AAATCCTGTGGTGGTCGT  R: AGATGCCCTTTGTGTGAGA |
| m-GBP2 | F: TTGAAGATGTTGAGAAGGGTGA  R: GCGGAATCGTCTACCCCAC |
| m-GBP3 | F: TTGTCTGGTGGAAAATTGG  R: CCCTTGGTTTCGGATTG |
| m-GBP4 | F: TTCCCTCCTCCACCTCTT  R: GAGACCCCACCCCTAGC |
| m-GBP5 | F: CCTGTCGAACTGCCATATT  R: TCATTGGTGCCTTCTTCC |
| m-GBP6 | F: AACACACTCCCTCTCCCA  R: GAAGCCAGTCAACATCCAG |
| m-GBP7 | F: CCTTCATGGACTGGGAGA  R: ATATCGGGTGTCATGTGGA |
| m-GAPDH | F: TGTGTCCGTCGTGGATCTGA  R: TTGCTGTTGAAGTCGCAGGAG |

Table S4. List of primary and secondary antibodies for Western blot analysis.

| Reagent | Source | Catalog number | Dilution |
| --- | --- | --- | --- |
| anti-GBP2 | Proteintech | 11854-1-AP | 1:1000 |
| anti-NIX | Cell Signaling Technology | D4R4B | 1:1000 |
| anti-TOM20 | Proteintech | 11802-1-AP | 1:10000 |
| anti-LC3 | Cell Signaling Technology | D11 | 1:1000 |
| anti-P62 | Proteintech | 18420-1-AP | 1:10000 |
| anti-FUNDC1 | Abclonal | A16318 | 1:1000 |
| anti-VDAC1 | Proteintech | 66345-1-Ig | 1:5000 |
| anti-Tyrosine Hydroxylase (TH) | Abcam | ab137869 | 1:5000 |
| anti-PINK1 | Proteintech | 23274-1-AP | 1:1000 |
| anti-HA-Tag | Cell Signaling Technology | C29F4 | 1:1000 |
| anti-MYC-Tag | Proteintech | 60003-2-Ig | 1:10000 |
| anti-DYKDDDDK (FLAG) Tag | Proteintech | 20543-1-AP | 1:50000 |
| anti-β-Actin | Proteintech | 66009-1-Ig | 1:10000 |
| HRP-conjugated Goat Anti-Mouse IgG(H+L) | Proteintech | SA00001-1 | 1:10000 |
| HRP-conjugated Goat Anti-Rabbit IgG(H+L) | Proteintech | SA00001-2 | 1:10000 |


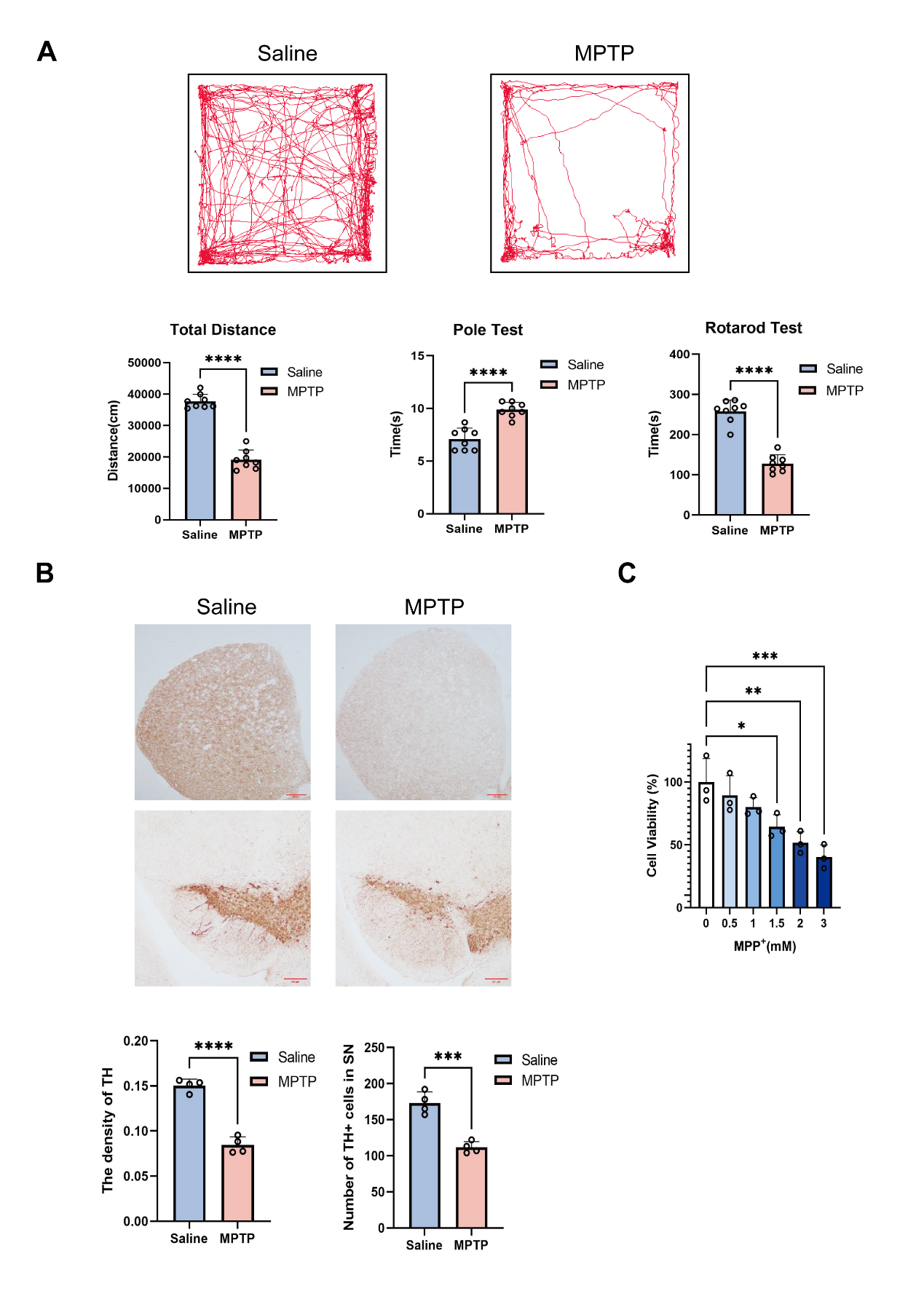


**Fig.S1.** Validation of the MPTP-induced mouse PD model and optimization of the MPP⁺-induced cellular PD model. (A and B) Successful induction of the PD mouse model was confirmed by (A) behavioral tests (n = 8) and (B) a significant loss of tyrosine hydroxylase (TH)-positive neurons in the substantia nigra, as assessed by immunohistochemistry (n = 4). (C) Cell viability of SH-SY5Y cells treated with varying concentrations of MPP⁺ was determined by CCK-8 assay (n = 3). A treatment of 1.5 mM MPP⁺ for 24 hours was selected for establishing the *in vitro* PD model in subsequent experiments.


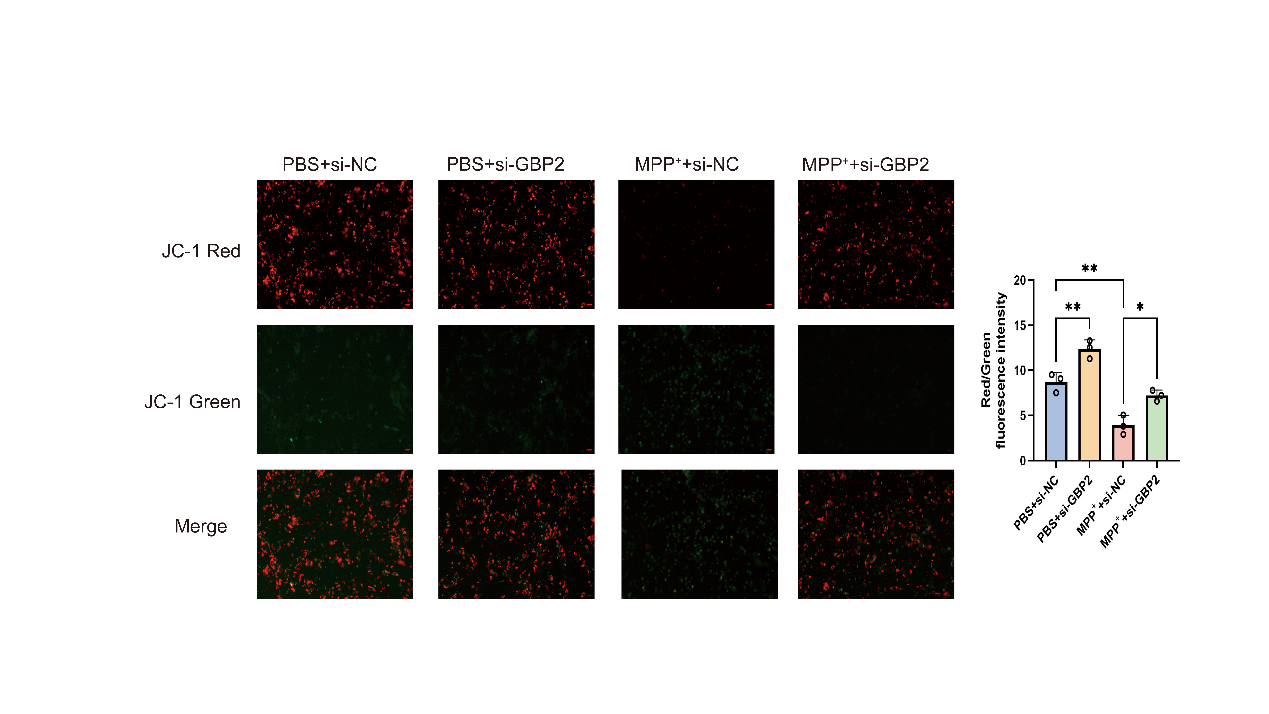


**Fig.S2.** The mitochondrial membrane potential was assessed by JC-1 staining in SH-SY5Y cells transfected with si-NC or si-GBP2 and treated with MPP⁺ or PBS (n=3 independent biological replicates).


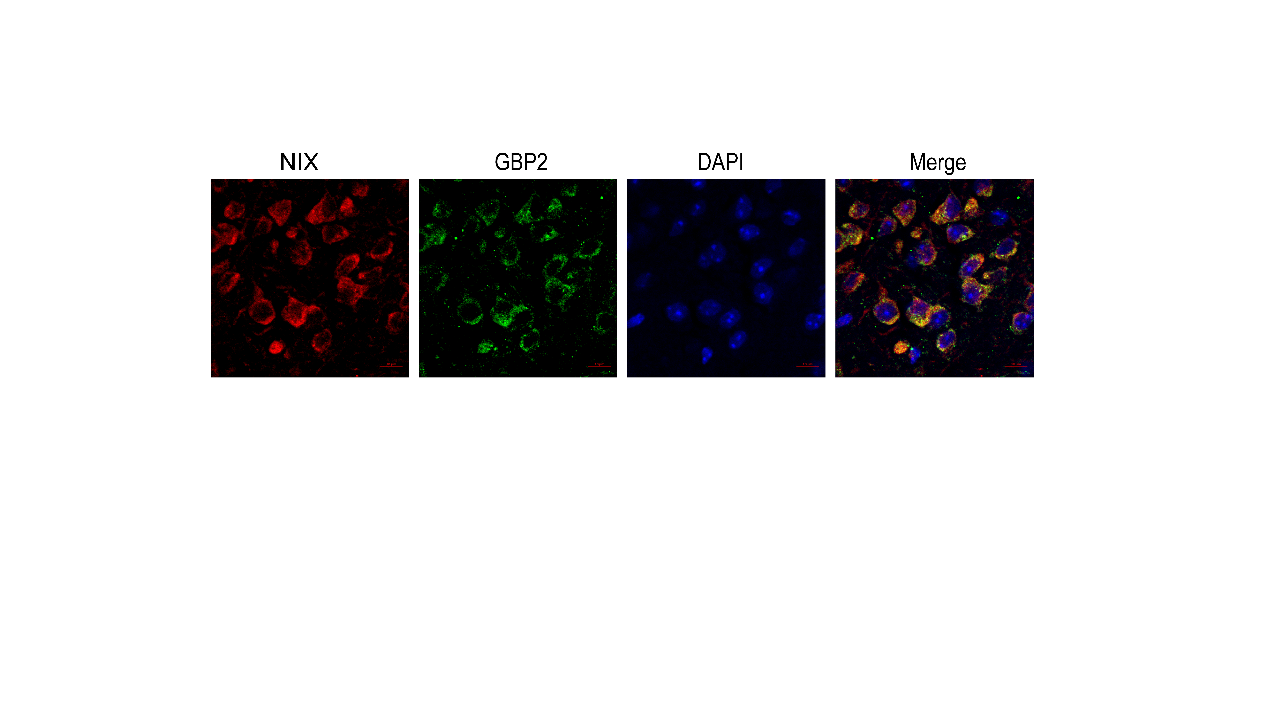


**Fig.S3.** Immunofluorescence confocal microscopy showing colocalization of endogenous GBP2 (green) and NIX (red) in the substantia nigra of mice. Scale bar, 10 μm.


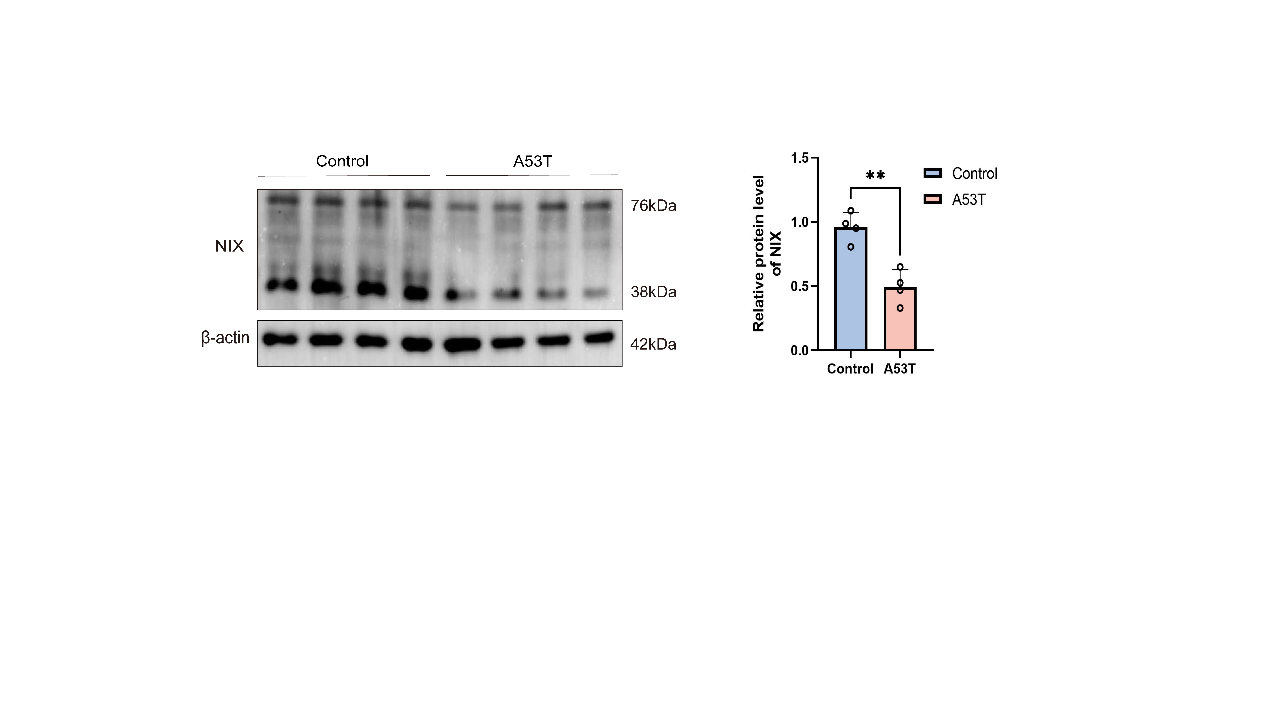


**Fig.S4.** Western blot showing NIX protein levels in the substantia nigra of A53T transgenic mice and control mice (n=4 per group). Data are presented as mean ± SEM. *p < 0.05, **p < 0.01, ***p < 0.001 for comparisons between the indicated groups.


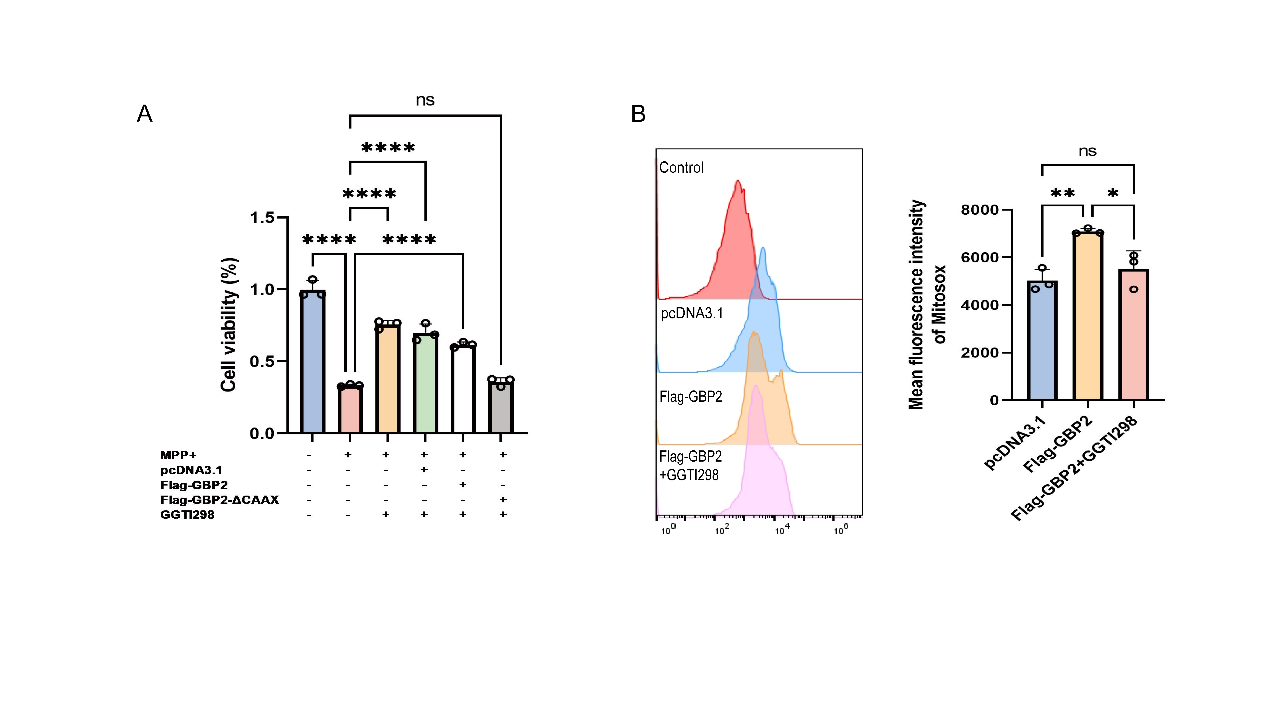


**Fig.S5.** (A) SH-SY5Y cells were transfected with Flag-GBP2, Flag-GBP2-ΔCAAX, or pcDNA3.1 (control), followed by treatment with GGTI298 and MPP⁺. Cell viability was significantly higher in the MPP⁺ + GGTI298 + Flag-GBP2 group compared to the MPP⁺ + GGTI298 + pcDNA3.1 group, while no significant change was observed in the Flag-GBP2-ΔCAAX group (n=3 independent biological replicates). (B) SH-SY5Y cells were transfected with Flag-GBP2 or pcDNA3.1 (control), followed by treatment with or without GGTI298. Mitosox level was significantly higher in the Flag-GBP2 group compared to the Flag-GBP2 + GGTI298 group (n=3 independent biological replicates).
